# Role of Zinc in Diabetic Kidney Disease

**DOI:** 10.3390/nu14071353

**Published:** 2022-03-24

**Authors:** Guido Gembillo, Luca Visconti, Alfio Edoardo Giuffrida, Vincenzo Labbozzetta, Luigi Peritore, Antonella Lipari, Vincenzo Calabrese, Giorgina Barbara Piccoli, Massimo Torreggiani, Rossella Siligato, Domenico Santoro

**Affiliations:** 1 Unit of Nephrology and Dialysis, Department of Clinical and Experimental Medicine, University of Messina, 98125 Messina, Italy; alfiogiuffrida91@libero.it (A.E.G.); vincenzo.labbozzetta@gmail.com (V.L.); luigiperitore1994@gmail.com (L.P.); anto.lipari19@gmail.com (A.L.); v.calabrese@outlook.it (V.C.); 2 Department of Biomedical and Dental Sciences and Morpho-Functional Imaging, University of Messina, 98125 Messina, Italy; rossellasiligato@gmail.com; 3 Unit of Nephrology and Dialysis, Ospedali Riuniti Villa Sofia Cervello, University of Palermo, 90146 Palermo, Italy; lucavisconti2003@yahoo.it; 4 Néphrologie Et Dialyse, Centre Hospitalier Le Mans, 194 Avenue Rubillard, 72000 Le Mans, France; gbpiccoli@yahoo.it (G.B.P.); mtorreggiani@ch-lemans.fr (M.T.); 5 Unit of Nephrology, Azienda Ospedaliera Universitaria Sant’Anna, 44124 Ferrara, Italy

**Keywords:** zinc, diabetic kidney disease, diabetic nephropathy, diabetes mellitus, antioxidant agent, chronic kidney disease

## Abstract

Diabetic Kidney Disease (DKD) represents the most common cause of Chronic Kidney Disease (CKD) in developed countries. Approximately 30% to 40% of diabetes mellitus (DM) subjects develop DKD, and its presence significantly increases the risk for morbidity and mortality. In this context, Zinc seems to have a potential role in kidney and body homeostasis in diabetic individuals as well as in patients at a high risk of developing this condition. This essential element has functions that may counteract diabetes-related risk factors and complications, which include stabilization of insulin hexamers and pancreatic insulin storage and improved glycemic control. In our review, we analyzed the current knowledge on the role of zinc in the management of renal impairment in course of DM. Several studies underline the critical role of zinc in reducing oxidative stress levels, which is considered the common denominator of the mechanisms responsible for the progression of kidney disease. Reaching and maintaining a proper serum zinc level could represent a valuable target to reduce symptoms related to DM complications and contrast the progression of kidney impairment in patients with the high risk of developing end-stage renal disease. In conclusion, analyzing the beneficial role of zinc in this review would advance our knowledge on the possible strategies of DM and DKD treatment.

## 1. Introduction

Zinc is an essential element and is the second most abundant divalent cation in the human body (2–4 g). It is mainly distributed in skeletal muscles (57%) and bones (29%) and acts as a cofactor for more than 300 enzymes, playing an important role in several biochemical pathways [1]. In addition, zinc is involved in the cellular mechanisms of proliferation, DNA and RNA synthesis, expression of specific genes, maintenance of structural integrity, and in the systemic regulation of the immune system [2,3]. Zinc deficiency is a frequent occurrence in the general population and is usually secondary to poor intake or absorption, and in some circumstances excessive loss [4]. In some studies, zinc deficiency has been reported to be associated with several disorders such as taste and growth alterations, increased risk of infections, and increased production of oxygen-free radicals [5]. In fact, zinc is essential for activating superoxide dismutase, a powerful enzyme with antioxidant activity [6].

Abnormalities of zinc homeostasis are frequent in patients with Chronic Kidney Disease (CKD). Plasma zinc levels decline progressively with decreasing GFR and, in hemodialysis patients, zinc deficiency reaches a prevalence between 40 and 78% [7]. Furthermore, a frequent complication of CKD is vascular calcification, which seems to be correlated with reduced zinc serum levels. In vitro studies suggested the protective effect of zinc in vascular calcification by enhancing vascular smooth muscle cell calcification viability in response to Hypoxia-inducible factor (HIF) stabilizers and high phosphate levels [8]. Voelkl et al. [9] evaluated the association between zinc levels and vascular calcifications in CKD patients, hemodialysis patients, and healthy volunteers. They showed that lower serum zinc levels correlated with increased vascular calcification in CKD patients, whereas zinc supplementation did not alter serum calcification propensity.

In addition, several studies have also indicated the possible interaction between zinc and diabetes and their associated complications, showing that physiologic zinc levels may have beneficial effects in diabetic patients [10]. In particular, diabetic kidney disease (DKD) is a frequent complication of diabetes mellitus that, in the early stages, manifests itself as microalbuminuria. Zinc’s role in reducing the incidence of kidney involvement in diabetes can be attributed to its ability in reducing oxidative stress and inflammation. Unfortunately, there are few studies evaluating the efficacy of zinc supplementation in preventing the onset of DKD, and most of them present controversial data.

The aim of this review is to examine the role of zinc in CKD and, in particular, its role in preventing the development of renal failure in diabetic patients and reducing the risk of related complications.

## 2. Zinc and Chronic Kidney Disease

CKD is a global public health problem with increasing prevalence worldwide, estimated to affect between 11 and 13% of the general population [11]. Reduced level of zinc has been demonstrated in CKD patients. Mafra et al. [12] assessed zinc status in 29 CKD patients who were not receiving dialysis, demonstrating an imbalance between higher erythrocytes zinc level (50.0 ± 7.2 microg/g hemoglobin) and borderline plasmatic titer (74 ± 17.7 microg/dL) compared with normal values. Tavares et al. performed a cross-sectional study with 21 nondialysis CKD patients compared with controls. They found that zinc plasma levels were significantly lower in CKD group compared with the control group (70.1 ± 19.2 μg/dL vs. 123.2 ± 24.6 μg/dL; *p* ˂ 0.0001) [13].

Another cross-sectional study conducted on 461 elderly Chinese individuals (> 90-year-old), including a group of CKD patients, showed that zinc levels were lower in the latter group and were negatively associated with the risk of CKD [14]. In end-stage renal disease (ESRD) patients receiving hemodialysis (HD) treatment, plasma zinc concentration appears to be even lower and often results in a true zinc deficiency. Hasanato et al. [15] studied 42 patients with ESRD on HD, 18 patients on peritoneal dialysis (PD), and 18 normal controls. No difference in plasma zinc was found between HD and PD patients (9.50 nmol/L; 95% CI: 7.83–12.09; IQR: 7.00–14.40) but both groups had lower titers than controls (13.20 nmol/L; 95% CI: 10.65–15.22; IQR: 10.58–15.35; *p* = 0.03). Lobo et al. [16] evaluated plasma zinc levels in 48 HD patients and 20 healthy subjects, confirming lower zinc levels in HD population (54.9 ± 16.1 μg/dL vs. 78.8 ± 9.4 μg/dL *p* < 0.05) and describing a negative correlation to both TNF-α (*p* = 0.0001) and LDL (*p* = 0.008) with a hypothesized correlation with higher cardiovascular mortality risk.

In a cross-sectional, case-control study, plasma zinc concentration was determined in 94 HD patients and compared with non-CKD patients, detecting a real zinc deficiency (serum Zn concentration < 70 μg/dL) in 57.83% of the HD group, in addition to the significant lower zinc titer in HD vs. control group (69.16 ± 17.29 μg/dL vs. 82.93 ± 14.75 μg/dL; *p* = 0.001) [17].

The mechanism by which zinc concentration is reduced in CKD patients is not entirely clear. Several factors may come into play. The reduced dietary intake appears to be related to protein restriction while poor gastrointestinal absorption could be secondary to 1,25-dihydroxycholecalciferol deficiency [18] or drug interactions such as phosphate binder [19]. Another possible cause of reduced zinc levels in CKD patients is increased urinary zinc excretion. Damianaki et al. [20] evaluated plasma and 24-h urinary zinc levels in 108 CKD patients compared with 81 healthy volunteers. The results showed that CKD patients had lower circulating zinc levels and higher 24-h urinary zinc excretion than control group (612.4 ± 425.9 mg/day vs. 479.2 ± 293.0 mg/day; *p* = 0.02) and fractional excretion (FE) of zinc was higher at more advanced CKD stages. Unfortunately, the cause of this disorder remains unknown. Recently, low zinc levels have been associated with an increased risk of developing CKD or progression of kidney disease.

Joo et al. [21] retrieved data from Korean Genome and Epidemiology Study and 7735 participants were included to assess the role of dietary zinc intake in the incidence of CKD. After a median follow-up of 11.5 years, CKD developed in 1409 (18.2%) participants. Cox regression analysis showed that the risk of developing CKD was higher in patients with lower zinc intakes (hazard ratio [HR], 1.36; 95% CI 1.18–1.58; *p* < 0.001).

In a retrospective cohort study, 325 CKD patients with zinc levels dosage were included and divided into two group: low zinc (plasma concentration < 60 μg/dL) and high zinc group (concentration ≥ 60 μg/dL). The primary outcome was defined as ESRD or death. The results showed that ESRD risk was higher in the low zinc group than in the high zinc group (43.1% vs. 20.4%, *p* < 0.001) but there was no difference in the risk of death (*p* = 0.65) [22].

Further, Damianaki et al. [20] found a significant correlation between lower zinc level and decline of kidney function in a cohort of 108 CKD patients compared with the healthy control group after 3 years follow-up. The pathogenic mechanism involving zinc levels in the decline of GFR is still unclear. Several studies report a key role of zinc in reducing oxidative stress levels that is considered the common denominator of all the mechanisms responsible for the progression of kidney disease [23].

Thus, zinc supplementation may be a useful resource in the future. In fact, it has been proposed not only to reduce the progression of kidney disease but also to reduce symptoms related to complications of ESRD in hemodialysis treatment.

A systematic review and meta-analysis evaluated the effect of zinc supplementation in HD patients, suggesting that it improves nutritional status and leads to an anti-inflammatory and antioxidative effect [24]. Kobayashi et al. [25] evaluated the effects of zinc supplementation on the response to erythropoietin-stimulating agents (ESA) in 35 HD patients with zinc deficiency (<65 μg/dL) compared with a control group of HD patients without supplementation at a 12-month follow-up, showing that the ESA dosage requirement was lower in the zinc group. In conclusion, there is a strong correlation between zinc levels and CKD and HD patients. Future studies are needed to evaluate the beneficial effects of zinc supplementation in these patients to reduce disease progression and the risk of developing comorbidity.

## 3. Role of the Zinc in DKD: Experimental Studies

The importance of zinc in preventing and slowing the progression of DKD has been widely evaluated in experimental studies, leading us to focus on this microelement and on the ways through which it exerts its protective action against the kidney damage sustained by diabetes mellitus.

One of the main pathogenic mechanisms lying under DKD onset is oxidative stress exerts by ROS in the kidney [26]. Ozcelik et al. [27] conducted an experimental study in rats with streptozotocin-induced diabetes. The group treated with zinc sulfate 30 mg/kg/day supplementation reduced the amount of kidney damage compared with the control group through the zinc-mediated activation of metallothionine, a protein rich in cysteine that interacts with Zn and iron bringing the reduction of reactive oxygen species (ROS) and, consequently, the oxidant process [28,29].

The protective effect of zinc administration was highlighted by the decrease in blood glucose level, microalbuminuria, and by a smaller amount of glomerular damage in renal samples of treated rats [28].

The relationship between zinc and DKD has been also demonstrated by Tang et al. [30] on diabetic rats supplemented with 5 mg/kg/day of zinc for 3 months. The supplementation decreased the expression of profibrotic elements such as connective tissue growth factor and upregulated the cardiac and renal metallothionines with an impact on both diabetic cardiopathy and DKD onset and progression, as demonstrated by the reduction of 24 h albuminuria and histological alterations.

In addition to metallothionine upregulation, other factors can be implicated in the zinc antioxidant mechanism [31]. One of these is nuclear factor-erythroid 2-related factor 2 (Nrf2). It is a powerful intracellular antioxidant ensuring an important defense against reactive oxygen species (ROS)-activating superoxide dismutase (SOD), glutathione S-transferase, and other neutralizing agents [32,33].

Zinc-mediated Nrf2 hyperexpression has been studied in several diseases [34,35] and in particular, in mice fibroblastic cells as shown by Mcmahon et al. [36], supporting the protective role of this mineral towards oxidative stress.

The study of Li et al. examined how zinc increased the transcription of Nrf2, attenuating DKD in vivo on diabetic experimental models and in vitro on human renal tubule cells [37]. Yang et al. [38] showed a protective role of zinc in diabetic rats through an increase in Nrf2, protein Kinase B, and glycogen synthase kinase 3 beta phosphorylation. These results were linked to downregulation of S0D1, SOD2, and other proteins involved in oxidative stress.

On the contrary, zinc deficiency leads to increased phlogistic processes and hyperexpression of intercellular adhesion molecule 1 (ICAM-1) in DKD.

Moreover, zinc can prevent the transition from epithelial to mesenchymal cells.

To confirm this action, a study on tubular cells of diabetic rats highlighted how zinc supplementation may reduce the risk of fibrosis modulating the expression of proteins such as E-cadherin and increase the expression of α-smooth muscle actin and vimentin [39]. Subsequently, Zhang et al. confirmed this zinc antifibrotic role, examining diabetic experimental models given 5 mg/kg zinc sulfate daily for 3 months. The attenuation of tubular-interstitial fibrosis was supported by the downregulation of hypoxia-inducible factor (HIF-1) [40], which is usually responsible for hypoxic damage in renal tubules having a fundamental role in epithelial-to-mesenchymal transition and in increasing the collagen matrix [41]. Another study demonstrated that zinc deficiency in diabetic mice increased albuminuria levels and led to hyperexpression of fibroblastic cells, likely increasing the transforming growth factor-β (TGF-β) concentrations, leading to the genesis and progression of tubular–interstitial fibrotic damage [42].

Another important mechanism through which zinc protects the kidney from chronic hyperglycemia damage is apoptosis. Through studies on cultures of mouse tubular cells, it has been shown that the zinc mineral reduces apoptosis by inhibiting caspase 3 and caspase 9, and the release of C-Cytochrome from mitochondria to cytosol [43]. The confirmation of these effects arrived by Wang et al. [44], who sustained the possible zinc anti-apoptotic role through the Nrf2-guided defensive process, which includes Wnt/β-catenin signaling pathway downregulation.

**Figure 1:**
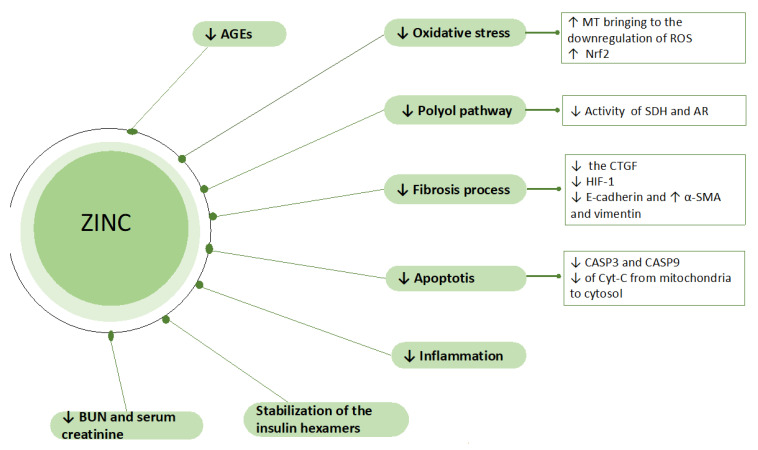
Zinc-related mechanisms in body homeostasis. AR, aldose reductase; CASP3, caspase 3; CASP9, caspase 9; CTGF, connective tissue growth factor; Cys-C, C-Cytochrome; HIF1, hypoxia inducible factor; MT, metallothionine; Nrf2, nuclear factor-erythroid 2-related factor 2; ROS, reactive oxygen species; SDH, sorbitol dehydrogenase; α-SMA, α-smooth muscle.

The Barman study group highlighted the importance of zinc supplementation in diabetic mice. The results obtained were an improvement in renal function calculated by creatinine clearance and a reduced oxidative stress decreasing levels of SOD, catalase, peroxidase glutathione, and glutathione S-transferase. In addition to these benefits, zinc has a demonstrated positive effect against the polyol pathway, reducing the enzymatic activity of sorbitol dehydrogenase and aldose reductase, and therefore, of their main metabolites—namely, sorbitol, fructose, and glucose. Furthermore, renal advanced glycation end products (AGEs) were diminished and the examined glomeruli demonstrated histological improvement (Figure 1) [45,46,47].

## 4. Role of the Zinc in DKD: Human Studies

DKD is the most common cause of CKD in developed countries. Approximately 30% to 40% diabetes mellitus patients develop DKD, and its presence significantly increases the risk for morbidity and mortality [48].

DKD is defined as the presence of persisting severely elevated albuminuria > 300 mg/24 h (or >200 μg/min) or an albumin-to-creatinine ratio (ACR) > 300 mg/g, confirmed in at least 2 of 3 samples, with concurrent presence of diabetic retinopathy and absence of signs of other forms of renal disease in both type 1 (T1D) and type 2 diabetes (T2D) [49].

The risk factors and predictors of development and progression of DKD include age at onset, hypertension, genetic, dietary habits, albuminuria, persistent hyperglycemia, dyslipidemia, obesity, smoking, race, the markers of inflammation and oxidation, serum advanced oxidation protein products (AOPP), AGEs, ROS, profibrotic cytokines (such as TGF-β), an increase in protein kinase C (PKC), and alterations of the metabolism of the polyols, among others [50,51,52].

Kidney biopsy is of pivotal importance for a correct diagnosis but also for defining prognosis, especially in the presence of heavy proteinuria or its rapid onset [53]. A timely correction of risk factors for DKD progression and a personalized therapeutic approach are fundamental in decreasing the impact of its complications and to reduce the risk of ESRD [54,55]. Different therapeutic approaches have been proposed for DKD treatment, including pharmaceutical and nutraceutical approaches [56,57,58]. Which is the best strategy for DKD prevention and management is still matter of debate [59,60].

In this context, Zinc seems to have a potential role in kidney and body homeostasis in diabetic individuals or for patients at a high risk to develop this condition. It seems to stabilize the insulin hexamers and insulin pancreatic storage, improve glycemia, and contrast diabetes-related risk factors [61,62].

Subjects following a diet with low zinc content or that present low levels of serum zinc present a major risk of cardiovascular diseases, DM onset, and developing glucose intolerance.

A recent systematic review and meta-analysis demonstrated that low-dose, long-duration zinc intake from supplements, and potentially biofortification, can benefit risk factors for T2DM and CVD [63].

In DKD setting, different studies demonstrated the connection between zinc serum levels and renal impairment progression (Table 1).

Xu et al. [64] demonstrated in a cohort of diabetic patients that serum zinc level is markedly lower, while urinary zinc level is significantly higher (*p* < 0.001) in both T1D and T2D subjects compared with controls.

The cross-sectional study of Al-Timini et al. [65] showed low serum zinc levels (*p* < 0.01) in DM subjects compared with non-DM controls. A significant decrease in eGFR (*p* < 0.05) and abnormal levels of microalbuminuria (*p* < 0.001) were reported in DM patients with low serum zinc level compared with those with normal serum zinc level. Serum zinc level in DM subjects was inversely correlated with serum creatinine (r = −0.331, *p* < 0.001) and microalbuminuria (r = −0.587, *p* < 0.001) and positively with e-GFR (r = 0.194, *p* < 0.01). In another cross-sectional study, Lin et al. [66] demonstrated that zinc status may be a marker of progression of CKD in T2D patients. Serum zinc levels presented a statistically significant decreasing trend (*p* trend = 0.032) from CKD stage 1 to stage 3b.

Several RCTs showed a pivotal role of zinc in preventing or slowing the worsening of renal function (Table 2).

Khan et al. [72] conducted a single-center, placebo-controlled RCT, studying the effects of 50 mg of zinc sulfate in addition to oral hypoglycemic agents and angiotensin converting enzyme inhibitors in subjects with T2D with microalbuminuria. Patients treated with zinc supplementation showed a significantly decrease in microalbumin, serum hs-CRP, mean fasting blood glucose, postprandial blood glucose, and glycosylated hemoglobin levels (*p* = 0.0001). Their results demonstrated that zinc supplementation can improve the oral hypoglycemic agents efficacy and reduce the risk complications of DKD.

These results are in line with those of Parham et al. [70] that analyzed the effect of zinc supplementation versus placebo on microalbuminuria in DM individuals in their double-blind, randomized, placebo-controlled, crossover trial.

The authors observed a significant reduction in albumin excretion in the zinc supplementation group but not in the placebo group from 86.5 ± 57 to 75 ± 71 mg/g (*p* = 0.01) and in the control group, after the switch from placebo to zinc compound (from 90 ± 60 mg/g to 85 ± 57 mg/g creatinine (*p* = 0.003) after 30 mg of elemental zinc supplementation.

In their randomized, double-blind, placebo-controlled clinical trial, Farvid et al. [67] investigated the effects of several minerals and vitamins on renal dysfunction in T2D. They studied the effects of the combination of these compounds in different groups of patients with similar characteristics; their results showed that the group treated with 200 mg vitamin C/day and 100 UI vitamin E/day and the group treated with zinc sulfate 30 mg/day and magnesium oxide 200 mg/day presented a decreased in urinary albumin excretion (*p* = 0.034 and *p* = 0.005, respectively), while the group treated only with zinc supplementation did not reach a significant reduction. One of the main limitations of this study is that ACR ratio was not searched in repeated measurements but only once in morning spot urine at baseline and after three months. This bias does not allow us to reach valid data and proper diagnosis of persistent microalbuminuria and macroalbuminuria.

This point has been also commented by Rossing et al. [67], who highlighted how this study showed a marked discrepancy between the level of urinary albumin excretion rate (30 mg/g creatinine), suggesting microalbuminuria, and the urinary protein excretion rate value (1–2 g/g creatinine; indicative of overt nephropathy).

Kadhim et al. [68], in their double-blind, placebo-controlled, clinical trial, studied the effects of 10 mg of melatonin and 50 mg of zinc acetate in addition to the regularly used metformin or placebo. Their results demonstrated that the combination of these micronutrients can significantly improve DM-related complications such as the impaired lipid profile and microalbuminuria in patients with T2D.

El-Ashmony et al. [71] studied the effects of 40 mg zinc sulphonate supplementation, in addition with oral hypoglycemic agents. Patients treated with zinc showed a significant reduction in blood urea nitrogen (BUN) and serum creatinine (*p* < 0.001), as well as better glycemic control and desirable changes in lipid profile.

Zinc supplementation has been demonstrated to exert a positive effect even in ESRD. In their randomized, double-blind, placebo-controlled trial, Hosseini et al. [68] tested the effects of zinc sulfate supplementation on DM patients on hemodialysis. The results of this study showed a favorable effect of 220 mg ZnSO_4_ (50 mg/day Zn) for 8 weeks supplementation on serum copeptin (*p* < 0.001), BUN (*p* < 0.001), and creatinine levels (*p* < 0.001) in hemodialysis subjects with zinc deficiency. In contrast, Quantitative Insulin Sensitivity Check Index (QUICKI) (*p* = 0.57), Homeostasis Model Assessment (HOMA-IR) (*p* = 0.60), and serum insulin (*p* = 0.55) were not influenced by zinc supplementation compared with placebo group.

## 5. Zinc Supplementation and Optimal Levels in DKD: In Medio Stat Virtus

The World Health Organization identifies zinc deficiency as a relevant contributing factor for major diseases [72]. Inadequate zinc levels are common even in developed countries but have devastating consequences, especially in low-income countries. Zinc deficiency is responsible for an estimated 453 207 deaths worldwide, 4.4% of which are among children 6–59 months of age and 1.0% of all-cause deaths [73].

The Biomarkers of Nutrition for Development Zinc Expert Panel and the International Zinc Nutrition Consultative Group suggest lower cut-offs for serum zinc between 59 and 70 µg/dL for females aged ≥10 years and between 61 and 71 µg/dL for males aged ≥10 years [74]. These cut-offs for zinc deficiency are in line with those from NHANES II study. In this survey, the low limits for zinc adequacy were 57–65 μg/dL for children <10 years, 61–74 μg/dL for males ≥10 years, and 59–70 μg/dL for females ≥10 years [75].

The Mayo Clinic Group established a normal range value for zinc plasma levels of 60–120 µg/dL for 0–10 year patients and of 66–110 µg/dL ≥ 11 years [76]. Ryan Wessels et al. [77], in their analysis, suggested maintaining plasma zinc levels within the physiologic range of ∼65–125 μg/dL, depending on age, sex, and fasting status.

The cause of suboptimal zinc levels can be related to different factors. Inadequate nutritional zinc intake is linked to inappropriate meat consumption, or secondary, to an excess of ingestions of food containing phytates or oxalates. Chronic diseases can also lead to inadequate zinc intake or excessive excretion [78,79]. Several medications can cause impaired zinc levels, such as penicillamine, diuretics, antibiotics, or sodium valproate [80]. 

The dietary intake of zinc for children is usually 3 mg/die, ranging between 8 to 11 mg/die for adults [81]. For adults with symptomatic zinc deficiency, oral replacement treatment usually comprehends a posology of 2 to 3 mg/kg/die for symptomatic subjects or 20–40 mg/die for 1–2 weeks. Doses higher than 50 mg/die can lead to gastric distress, nausea, headache, loss of appetite, and diarrhea. A supplementation higher than 150 mg/die can lead to immune alterations and lipidic status dysregulation [82].

In diabetic patients, zinc seems to exert several positive effects. Zinc supplementation in T2D subjects could ameliorate HbA1c% levels, fasting, and postprandial glucose [83,84]. A recent systematic review and meta-analysis showed that moderately high dietary zinc intake, concerning the Dietary Reference Intake, could reduce the risk of T2D by 13%, and up to 41% in rural areas. Conversely, elevated plasma zinc concentration was associated with an increased risk of T2D by 64%, indicating alterations in zinc homeostasis [85].

It is still a matter of debate as to which is the possible optimal serum zinc value for diabetic patients with kidney impairment to slow the disease progression. This population often exhibits impairment of plasma zinc levels with hyperzincuria and altered zinc reabsorption compared with the general population [86,87].

A population-based reference value for zinc in DKD patients is still missing and further studies are needed to establish its optimal level. At the same time, the existing literature on the field suggests maintaining an adequate status of this microelement, avoiding low critical levels or, conversely, an excessive supplementation.

## 6. A Tricky Aspect on the Zinc Supplementation: Mind the Copper

One of the limitations of the current studies on zinc is the lack of data about the balance between zinc and copper. Zinc is essential for the adequate formation and function of the antioxidant enzyme copper–zinc superoxide dismutase, and a proper balance between these two micronutrients is of pivotal importance in body homeostasis.

Reduced blood concentrations of zinc and copper cause a significant number of diseases, ranging from growth retardation to immunosuppression, to syndromes characterized by anemia, neutropenia and skeletal alterations. In addition, zinc and copper reduction alters the patient’s lipid profile, activating atherogenesis with a consequent increase in cardiovascular risk. Interestingly, it has been observed that detecting low levels of zinc and its subsequent supplementation can reduce copper, with rather important consequences. Fischer et al. [88] observed how the reduction in copper and zinc concentrations caused a reduced activity of metalloenzymes and ceruloplasmin. However, the administration of zinc with two doses of 2.5 mg per day showed no statistically significant differences between the treated group and the control group at 2–4 and 6 weeks after starting therapy. SOD activity was even reduced in treated patients compared with the control group, with a significant difference between the groups at 6 week (*p* < 0.05). These results indicated that the zinc supplements decreased the copper status of the experimental group.

Koi et al. [89] confirmed the hypothesis of the inverse relationship between zinc supplementation and blood copper reduction. They reported a clinical case of a 75-year-old patient treated with hemodialysis for 10 years, with kidney damage caused by DKD. The patient, in treatment with zinc supplements, after about 3 months, developed pancytopenia with high serum levels of WT1 mRNA and the presence of megaloblasts and ring sideroblasts at the bone marrow needle aspiration exam. These results, associated with low serum levels of copper and ceruloplasmin, suggested that the cause of these hematopoietic alterations was attributable to zinc supplements therapy and a consequent reduction in cupremia values. To demonstrate this hypothesis, the patient added cocoa (rich in copper) to his diet in the following months, gradually solving pancytopenia and bone marrow dysplasia.

Duncan et al. [90] demonstrated how supplementation with zinc, in subjects with inadequate serum levels, induced a copper deficiency. In their study, 62% of the receiving zinc supplementation experienced copper reduction. In 48% of the studied population, the cause of zinc low titer was attributable to reduced albumin concentration, and a persistent systemic inflammatory state. Nine percent of patients also developed unexplained anemia and seven percent presented typical neurological symptoms of copper deficiency.

In a recent study, Takao et al. [67] evaluated the role of inflammation markers and the copper/zinc ratio in developing DKD in patients with T2D. The authors noticed that an increase in this ratio may further exacerbate inflammation in patients with elevated soluble tumor necrosis factor-α receptor 1; moreover, this inflammatory status, which correlates with an increase in copper/zinc ratio levels, is synergistically associated with a high prevalence of DKD.

Closely related to this study is that of Laouali et al. [91], in which the incidence of diet on the serum Cu/Zn ratio was evaluated, and the consequent development of T2D in women. A total 70,991 women were followed for 20 years, calculating the amounts of zinc and copper taken with the diet through questionnaires. In this context, Cu/Zn ratio < 0.55 was associated with a lower risk of developing T2D. For women with zinc intake <8 mg/day, a higher Cu/Zn ratio was associated with a higher T2D risk.

Subgroups of patients were analyzed, comparing the highest and lowest quintiles of the Cu/Zn ratio and the same pattern of association was observed with obese women or taking more than 8 mg of zinc a day. For women with zinc intake <8 mg/day, higher Cu/Zn ratio was associated with higher T2D risk. The study suggests that a lower dietary Cu/Zn ratio is associated with a lower T2D risk, especially among obese women and women with zinc intake >8 mg/day.

## 7. Conclusions

Zinc is a fundamental microelement involved in essential pathways regulating body homeostasis. Zinc supplementation seems to exert a favorable effect not only on DKD risk factors but also on delaying its progression. Several cross-sectional studies and RCTs confirmed that imbalances in zinc levels lead to high susceptibility to the development of DM and its complications.

An adequate level of this essential compound can be fundamental in contrast oxidative stress and systemic inflammation.

Even if several studies seem to demonstrate that zinc supplementation may have renoprotective effects in patients with DKD, more RCTs on larger patients’ sample and with longer duration of follow-up are needed to confirm the effects of long-term zinc treatment and the correct balance of copper/zinc status to evaluate the appropriate supplementation dosage.

## Figures and Tables

**Table 1:** Cross-sectional studies analyzing zinc serum levels and renal impairment progression in DKD. Abbreviations: CKD, Chronic kidney disease; Cu, Copper; DKD, Diabetic Kidney Disease; T1D, Type 1 diabetes mellitus; T2D, Type 2 diabetes mellitus; DKD, Diabetic Kidney Disease; DPN, Diabetic peripheral neuropathy; DR, Diabetic retinopathy; eGFR, estimated glomerular filtration rate; IFG, Impaired fasting glucose; IGT, Impaired glucose tolerance; PPM, part per million; Zn, Zinc.

Study, year	Patients (*n*.)	Controls (*n*.)	Results
Jiancheng Xu et al. [64] 2013	189 patients with DM or prediabetes Age 20–65, mean age 55 IFG: (*n* = 12) IGT: (*n* = 15) T1D: (*n* = 25) T2D: (*n* = 137) DKD: (*n* = 24) DR: (*n* = 34) DPN: (*n* = 50)	50 healthy patients	Plasma Zn (mg/L): Control group 0.81 (0.67–0.93) vs. IFG group 0.75 (0.70–0.84) (NS) vs. IGT 0.77 (0.67–0.87) (NS) vs. T1D 0.59 (0.53–0.75) (*p* = 0.056) vs. T2D 0.61 (0.51–0.75) (*p* < 0.001) Urinary Zn (mg/L): Control 0.20 (0.14–0.32) vs. IFG 0.32 (0.26–0.37) (NS) vs. IGT 0.27 (0.19–0.41) (NS) vs. T1D 0.86 (0.67–0.91) *p* < 0.001 vs. T2D 0.48 (0.38–0.57) *p* < 0.001 Plasma Zn (mg/L): T2D 0.73 (0.55–0.79) vs. DKD 0.59 (0.48–0.76) (NS) vs. DR 0.58 (0.46–0.63) *p* = 0.002 vs. DPN 0.63 (0.59–0.75) *p* = 0.08 Urinary Zn (mg/L): T2D 0.47 (0.28–0.53) vs. DKD 0.44 (0.30–0. 52) (NS) vs. DR 0.45 (0.25–0.52) (NS) vs. DPN 0.52 (0.44–0.63) (*p* < 0.001)
Al Timini et al. [65] 2014	300 T2D patients Age 43.5–71.6 Group II: Diabetic, normotensive (*n* = 145) Group III: Diabetic, hypertensive (*n* = 41) Group IV: Diabetic, normotensive with microalbuminuria (*n* = 62) Group V: Diabetic, hypertensive with microalbuminuria (*n* = 52)	100 apparently healthy subjects Age 45.7–69.2 Group I: Nondiabetic, normotensive (*n* = 100)	Urinary zinc/creatine (ug/g): 2.33 + 1.18 in patient groups vs. 1.01 + 0.57 in control (*p* value < 0.001) Serum zinc (ug/dL): 70.0 + 19.2 in patient groups vs. 86.2 + 15.2 in control (*p* value < 0.001) Group I: 86.2 + 15.2 Group II: 79.2 + 15.0 Group III: 77.9 + 17.2 Group IV: 56.8 + 13.8 Group V: 55.0 + 14.2 eGFR > 90 mL/min/1.73 m^2^: 107 (45.7%) had serum zinc levels < 70 ug/dL and 127 (54.3%) > 70 ug/dL (total 234) eGFR 60–89 mL/min/1.73 m^2^: 38 (76.0%) had serum zinc levels < 70 ug/dL and 12 (24.0%) > 70 ug/dL (total 50) eGFR 30–59 mL/min/1.73 m^2^: 14 (93.3%) had serum zinc levels < 70 ug/dL and 1 (6.7%) > 70 ug/dL (total 15) eGFR 15–29 mL/min/1.73 m^2^: 1 (100%) had serum zinc levels < 70 μg/dL and none > 70 μg/dL (total 1)
Lin et al. [66] 2018	148 T2D patients with CKD Age 62.4 ± 9.8 CKD Stage 1 eGFR > 90 mL/min/1.73 m^2^ (*n* = 25) CKD Stage 2 eGFR 60–89 mL/min/1.73 m^2^ (*n* = 49) CKD Stage 3a eGFR 45–69 mL/min/1.73 m^2^ (*n* = 40) CKD Stage 3b eGFR 30–44 mL/min/1.73 m^2^ (*n* = 34	No control group	Zinc (ppm) Stage 1: 1.0 ± 0.2 Stage 2: 0.9 ± 0.2 Stage 3a: 0.9 ± 0.2 Stage 3b: 0.8 ± 0.2 *p* = <0.001
Takao et al. [67] 2021	651 patients with T2D Age 65 ± 9.7 DKD group (*n* = 220) No DKD group (*n* = 431)	No control group	Cu (microg/dL): 97.0 ± 15.6 in no DKD group vs. 100.5 ± 15.5 in DKD group (*p* = 0.007) Zn (microg/dL): 85.4 ± 11.3 in no DKD group vs. 82.1 ± 11.6 in DKD group (*p* = 0.0005) Cu/Zn ratio: 1.155 ± 0.242 in no DKD group vs. 1.247 ± 0.265 in DKD group (*p* < 0.0001) The optimal Cu\Zn cut-off value for detecting DKD was 1.1648

**Table 2:** Randomized-Controlled Trials analyzing zinc serum levels and renal impairment progression in DKD. Abbreviations: NAG, N-Acetyl-β-d-Glucosaminidase; HDL, High-Density Lipoprotein; VLDL, Very-Low-Density Lipoprotein; LDL, Low-Density Lipoprotein; SBP, Systolic Blood Pressure; DBP, Diastolic Blood Pressure; OHA, Oral Hypoglycemic Agents; UAE, *urinary* albumin excretion; FBG, fasting blood glucose; TG, triglycerides; CRP; C-reactive protein.

Study, Year	Population	Intervention	Control	Follow-Up	Renal Outcomes
Farvid et al. [68] 2005	76 T2D patients with microalbuminuria Divided into 4 groups: M, V, MV, P Age: 50 ± 9 for groups P, V, MV 52 ± 8 for group M	Group M (*n* = 18): Zinc sulfate 15 mg and magnesium oxide 100 mg twice a day Group V (*n* = 20): 100 mg vitamin C and 50 UI vitamin E twice a day Group MV (*n* = 19): Both supplementation of group M and V	Group P (*n* = 19): Placebo	3 months	Microalbuminuria (mg/g creatinine): No significative reduction after zinc supplementation in group P and M Significative reduction in group V (35.6 (6.2–64.9) vs. 22.1 (5.2–39.0) (*p* = 0.034) and in group MV (29.3 (3.2 to 61.9) vs. 10.8 (4.2–17.3) (*p* = 0.005) after zinc supplementation NAG (units/g Cr): No significative reduction after zinc supplementation in group M, V, MV, and P Urine protein (g/g creatinine): No significative reduction after zinc supplementation in group M, V, MV, and P
Kadhim et al. [69] 2006	46 patients with T2D Divided into 3 groups: A, B, C, and control group Aged 49.1 ± 6.0	Group B (*n* = 18): 10 mg of melatonin + 50 mg of zinc acetate + metformin 2550 mg/die + dietary control program Group C (*n* = 13): 10 mg of melatonin + 50 mg of zinc acetate + dietary control program	Group A (*n* = 15): Placebo + metformin 2550 mg/die Control (*n* = 17): healthy subjects in the same age range of patients	3 months	Microalbuminuria (mg/g creatinine): No significative difference in group A after Zinc supplementation Significative reduction in group B after 30 days and after 90 days Baseline 249.05 ± 24.74. After 30 days 188.61 ± 13.88 (*p* < 0.01). After 90 days 156.22 ± 14.26 (*p* < 0.01). Significative difference between group B and group A after 90 days. Significative reduction in group C after 30 days and after 90 days. Baseline 254.69 ± 32.30. After 30 days 215.07 ± 30.96 (*p* < 0.01). After 90 days 621.07 ± 18.59 (*p* < 0.05). Significative difference between group C and group A after 90 days Plasma creatinine (mg/dL): No significative difference in group A, group B, and group C after Zinc supplementation Plasma urea (mg/dL): No significative difference in group A, group B, and group C after Zinc supplementation
Parham et al. [70] 2008	50 patients with T2D and microalbuminuria Divided into group 1 (*n* = 21) and group 2 (*n* = 18) Age 52 ± 9.3 (Group 1) and 54.5 ± 9.2 (Group 2)	Zinc Sulfate 132 mg 1 capsule per day (30 mg elemental Zn)	Placebo (30 mg of lactose)	Group 1: 3 months of zinc, 4 weeks of wash out, and 3 months of placebo Group 2: 3 months of placebo, 4 weeks of wash out, and 3 months of zinc	Urinary albumin excretion (mg/g): Significant reduction after 3 months of Zn supplementation in both Group 1 (86.5 ± 57 at baseline vs. 75 ± 71 after supplementation) and Group 2 (90 ± 60 at baseline vs. UAE = 78 ± 57 after supplementation) (*p* < 0.05) No significant reduction in both group after placebo Creatinin clearance (mL/min/1.73 m^2^): No significant difference in both groups concerning Creatinine Clearance GFR (mL/min/1.73 m^2^) No significant difference in both groups concerning GFR
El-Ashmony et al. [71] 2012	Patients with T2D Age: 48.46 ± 4.61 in Zn Group and 48.20 ± 4.09 in placebo group. Patients on regular use of antidiabetic drugs (no insuline), with HB1Ac concentration of 8% or greater	Zn group (*n* = 26) 40 mg zinc sulfate once daily	Control group (*n* = 30) Placebo	8 weeks	BUN (mg/dL): Zn group: before treatment 24.15 ± 6.28 and 21.15 ± 6.04 after treatment (*p <* 0.001) No significative difference in control group Serum creatinine (mg/dL): Zn group: before treatment 0.90 ± 0.42 and 0.82 ± 0.42 after treatment (*p <* 0.001) No significative difference in control group
Khan et al. [72] 2013	54 T2D patients with microalbuminuria divided into 2 groups: OHA alone or OHA + zinc. Age 56.1 ± 8.5	Zn group (*n* = 27) Zinc Sulfate 50 mg 1 capsule per day + OHA	Control group (*n* = 27) OHA alone	12 weeks	Urine microalbumin (mg/day): Significant reduction after 12 weeks in Zinc group Pretrial: 146.87 ± 30.83 before treatment vs. 80.70 ± 33.99 after treatment *p* value = <0.0001 No reduction in control group

## Data Availability

Not applicable.

## Abbreviations

AOPP	advanced oxidation protein products
ACR	albumin-to-creatinine ratio
AGEs	advanced glycation end products
BUN	blood urea nitrogen
CKD	Chronic Kidney Disease
DM	Diabetes Mellitus
DKD	Diabetic Kidney Disease
ESRD	end-stage renal disease
Fe	iron
FE	fractional excretion
GFR	glomerular filtration rate
HD	hemodialysis
HIF	Hypoxia-inducible factor
HOMA-IR	homeostasis model assessment—insulin resistance
hs-CRP	high-sensitivity c-reactive protein
HR	hazard ratio
HIF	Hypoxia-inducible factor
ICAM-1	intercellular adhesion molecule 1
LDL	low-density lipoprotein
MT	metallothionine
mRNA	messenger ribonucleic acid
Nrf2	nuclear factor-erythroid 2-related factor 2
PD	peritoneal dialysis
QUICKI	Quantitative Insulin Sensitivity Check Index
RCT	randomized controlled trial
ROS	reactive oxygen species
SOD	superoxide dismutase
PKC	protein kinase c
TGF-β	Transforming growth factor β
TNF-α	tumor necrosis factor-alpha
T2DM	type 2 diabetes mellitus
WT1	Will’s tumor gene-1
Zn	Zinc
ZnSO_4_	Zinc sulfate

## References

[1] King J.C., Shames D.M., Woodhouse L.R. (2000). Zinc homeostasis in humans. J. Nutr..

[2] Mac Donald R.S. (2000). The role of zinc in growth and cell proliferation. J. Nutr..

[3] Prasad A.S. (2009). Zinc: Role in immunity, oxidative stress and chronic inflammation. Curr. Opin. Clin. Nutr. Metab. Care.

[4] Prasad A.S. (2012). Discovery of human zinc deficiency: 50 years later. J. Trace Elem. Med. Biol..

[5] Prasad A.S. (1985). Clinical manifestations of zinc deficiency. Annu. Rev. Nutr..

[6] Prasad A.S., Bao B. (2019). Molecular Mechanisms of Zinc as a Pro-Antioxidant Mediator: Clinical Therapeutic Implications. Antioxidants.

[7] Dvornik S., Cuk M., Racki S., Zaputović L. (2006). Serum zinc concentrations in the maintenance hemodialysis patients. Coll. Antropol..

[8] Nagy A., Pethő D., Gáll T., Zavaczki E., Nyitrai M., Posta J., Zarjou A., Agarwal A., Balla G., Balla J. (2020). Zinc Inhibits HIF-Prolyl Hydroxylase Inhibitor-Aggravated VSMC Calcification Induced by High Phosphate. Front. Physiol..

[9] Voelkl J., Tuffaha R., Luong T.T.D., Zickler D., Masyout J., Feger M., Verheyen N., Blaschke F., Kuro O.M., Tomaschitz A. (2018). Zinc Inhibits Phosphate-Induced Vascular Calcification through TNFAIP3-Mediated Suppression of NF-κB. J. Am. Soc. Nephrol..

[10] Ranasinghe P., Pigera S., Galappatthy P., Katulanda P., Constantine G.R. (2015). Zinc and diabetes mellitus: Understanding molecular mechanisms and clinical implications. DARU.

[11] Hill N.R., Fatoba S.T., Oke J.L., Hirst J.A., O’Callaghan C.A., Lasserson D.S., Hobbs F.D. (2016). Global Prevalence of Chronic Kidney Disease—A Systematic Review and Meta-Analysis. PLoS ONE.

[12] Mafra D., Cuppari L., Cozzolino S.M. (2002). Iron and zinc status of patients with chronic renal failure who are not on dialysis. J. Ren. Nutr..

[13] Tavares A.P.D.S.R., Mafra D., Leal V.O., Gama M.D.S., Vieira R.M.M.F., Brum I.S.D.C., Borges N.A., Silva A.A. (2021). Zinc Plasma Status and Sensory Perception in Nondialysis Chronic Kidney Disease Patients. J. Ren. Nutr..

[14] Shen Y., Yin Z., Lv Y., Luo J., Shi W., Fang J., Shi X. (2020). Plasma element levels and risk of chronic kidney disease in elderly populations (≥90 Years old). Chemosphere.

[15] Hasanato R.M. (2014). Assessment of trace elements in sera of patients undergoing renal dialysis. Saudi Med. J..

[16] Lobo J.C., Stockler-Pinto M.B., Farage N.E., Faulin Tdo E., Abdalla D.S., Torres J.P., Velarde L.G., Mafra D. (2013). Reduced plasma zinc levels, lipid peroxidation, and inflammation biomarkers levels in hemodialysis patients: Implications to cardiovascular mortality. Ren. Fail..

[17] Dashti-Khavidaki S., Khalili H., Vahedi S.M., Lessan-Pezeshki M. (2010). Serum zinc concentrations in patients on maintenance hemodialysis and its relationship with anemia, parathyroid hormone concentrations and pruritus severity. Saudi J. Kidney Dis. Transpl..

[18] Kambe T., Tsuji T., Hashimoto A., Itsumura N. (2015). The Physiological, Biochemical, and Molecular Roles of Zinc Transporters in Zinc Homeostasis and Metabolism. Physiol. Rev..

[19] Takagi K., Masuda K., Yamazaki M., Kiyohara C., Itoh S., Wasaki M., Inoue H. (2010). Metal ion and vitamin adsorption profiles of phosphate binder ion-exchange resins. Clin. Nephrol..

[20] Damianaki K., Lourenco J.M., Braconnier P., Ghobril J.P., Devuyst O., Burnier M., Lenglet S., Augsburger M., Thomas A., Pruijm M. (2020). Renal handling of zinc in chronic kidney disease patients and the role of circulating zinc levels in renal function decline. Nephrol. Dial. Transplant..

[21] Joo Y.S., Kim H.W., Lee S., Nam K.H., Yun H.R., Jhee J.H., Han S.H., Yoo T.H., Kang S.W., Park J.T. (2021). Dietary zinc intake and incident chronic kidney disease. Clin. Nutr..

[22] Tokuyama A., Kanda E., Itano S., Kondo M., Wada Y., Kadoya H., Kidokoro K., Nagasu H., Sasaki T., Kashihara N. (2021). Effect of zinc deficiency on chronic kidney disease progression and effect modification by hypoalbuminemia. PLoS ONE.

[23] Li M.S., Adesina S.E., Ellis C.L., Gooch J.L., Hoover R.S., Williams C.R. (2017). NADPH oxidase-2 mediates zinc deficiency-induced oxidative stress and kidney damage. Am. J. Physiol. Cell Physiol..

[24] Wang L.J., Wang M.Q., Hu R., Yang Y., Huang Y.S., Xian S.X., Lu L. (2017). Effect of Zinc Supplementation on Maintenance Hemodialysis Patients: A Systematic Review and Meta-Analysis of 15 Randomized Controlled Trials. Biomed. Res. Int..

[25] Kobayashi H., Abe M., Okada K., Tei R., Maruyama N., Kikuchi F., Higuchi T., Soma M. (2015). Oral zinc supplementation reduces the erythropoietin responsiveness index in patients on hemodialysis. Nutrients.

[26] Pan H.Z., Zhang L., Guo M.Y., Sui H., Li H., Wu W.H., Qu N.Q., Liang M.H., Chang D. (2010). The oxidative stress status in diabetes mellitus and diabetic nephropathy. Acta Diabetol..

[27] Özcelik D., Nazıroglu M., Tunçdemir M., Çelik Ö., Öztürk M., Flores-Arce M.F. (2012). Zinc supplementation attenuates metallothionein and oxidative stress changes in kidney of streptozotocin-induced diabetic rats. Biol. Trace Elem. Res..

[28] Zhou Z., Sun X., Kang Y.J. (2002). Metallothionein protection against alcoholic liver injury through inhibition of oxidative stress. Exp. Biol. Med..

[29] Cai L. (2004). Metallothionein as an adaptive protein prevents diabetes and its toxicity. Nonlinearity Biol. Toxicol. Med..

[30] Tang Y., Yang Q., Lu J., Zhang X., Suen D., Tan Y., Jin L., Xiao J., Xie R., Rane M. (2010). Zinc supplementation partially prevents renal pathological changes in diabetic rats. J. Nutr. Biochem..

[31] Apostolova M.D., Choo K.H., Michalska A.E., Tohyama C. (1997). Analysis of the possible protective role of metallothionein in streptozotocin-induced diabetes using metallothionein-null mice. J. Trace Elem. Med. Biol..

[32] He X., Kan H., Cai L., Ma Q. (2009). Nrf2 is critical in defense against high glucose-induced oxidative damage in cardiomyocytes. J. Mol. Cell Cardiol..

[33] Tan Y., Ichikawa T., Li J., Si Q., Yang H., Chen X., Goldblatt C.S., Meyer C.J., Li X., Cai L. (2011). Diabetic downregulation of Nrf2 activity via ERK contributes to oxidative stress-induced insulin resistance in cardiac cells in vitro and in vivo. Diabetes.

[34] Kim H.J., Vaziri N.D. (2010). Contribution of impaired Nrf2-Keap1 pathway to oxidative stress and inflammation in chronic renal failure. Am. J. Physiol. Renal. Physiol..

[35] Mehta A.J., Joshi P.C., Fan X., Brown L.A., Ritzenthaler J.D., Roman J., Guidot D.M. (2011). Zinc supplementation restores PU.1 and Nrf2 nuclear binding in alveolar macrophages and improves redox balance and bacterial clearance in the lungs of alcohol-fed rats. Alcohol. Clin. Exp. Res..

[36] McMahon M., Lamont D.J., Beattie K.A., Hayes J.D. (2010). Keap1 perceives stress via three sensors for the endogenous signaling molecules nitric oxide, zinc, and alkenals. Proc. Natl. Acad. Sci. USA.

[37] Li B., Cui W., Tan Y., Luo P., Chen Q., Zhang C., Qu W., Miao L., Cai L. (2014). Zinc is essential for the transcription function of Nrf2 in human renal tubule cells in vitro and mouse kidney in vivo under the diabetic condition. J. Cell Mol. Med..

[38] Yang F., Li B., Dong X., Cui W., Luo P. (2017). The beneficial effects of zinc on diabetes-induced kidney damage in murine rodent model of type 1 diabetes mellitus. J. Trace Elem. Med. Biol..

[39] Zhang X., Liang D., Chi Z.H., Chu Q., Zhao C., Ma R.Z., Zhao Y., Li H. (2015). Effect of zinc on high glucose-induced epithelial-to-mesenchymal transition in renal tubular epithelial cells. Int. J. Mol. Med..

[40] Zhang X., Liang D., Fan J., Lian X., Zhao Y., Wang X., Chi Z.H., Zhang P. (2016). Zinc Attenuates Tubulointerstitial Fibrosis in Diabetic Nephropathy Via Inhibition of HIF Through PI-3K Signaling. Biol. Trace Elem. Res..

[41] Sun S., Ning X., Zhang Y., Lu Y., Nie Y., Han S., Liu L., Du R., Xia L., He L. (2009). Hypoxia-inducible factor-1alpha induces Twist expression in tubular epithelial cells subjected to hypoxia, leading to epithelial-to-mesenchymal transition. Kidney Int..

[42] Zhang X., Liang D., Lian X., Chi Z.H., Wang X., Zhao Y., Ping Z. (2016). Effect of zinc deficiency on mouse renal interstitial fibrosis in diabetic nephropathy. Mol. Med. Rep..

[43] Zhang X., Zhao Y., Chu Q., Wang Z.Y., Li H., Chi Z.H. (2014). Zinc modulates high glucose-induced apoptosis by suppressing oxidative stress in renal tubular epithelial cells. Biol. Trace Elem. Res..

[44] Wang S., Nie P., Lu X., Li C., Dong X., Yang F., Luo P., Li B. (2020). Nrf2 participates in the anti-apoptotic role of zinc in Type 2 diabetic nephropathy through Wnt/β-catenin signaling pathway. J. Nutr. Biochem..

[45] Barman S., Pradeep S.R., Srinivasan K. (2018). Zinc supplementation alleviates the progression of diabetic nephropathy by inhibiting the overexpression of oxidative-stress-mediated molecular markers in streptozotocin-induced experimental rats. J. Nutr. Biochem..

[46] Li B., Tan Y., Sun W., Fu Y., Miao L., Cai L. (2013). The role of zinc in the prevention of diabetic cardiomyopathy and nephropathy. Toxicol. Mech. Methods.

[47] Karatug A., Kaptan E., Bolkent S., Mutlu O., Yanardag R. (2013). Alterations in kidney tissue following zinc supplementation to STZ-induced diabetic rats. J. Trace Elem. Med. Biol..

[48] Bonner R., Albajrami O., Hudspeth J., Upadhyay A. (2020). Diabetic Kidney Disease. Prim Care.

[49] Selby N.M., Taal M.W. (2020). An updated overview of diabetic nephropathy: Diagnosis, prognosis, treatment goals and latest guidelines. Diabetes Obes. Metab..

[50] Conti G., Caccamo D., Siligato R., Gembillo G., Satta E., Pazzano D., Carucci N., Carella A., Campo G.D., Salvo A. (2019). Association of Higher Advanced Oxidation Protein Products (AOPPs) Levels in Patients with Diabetic and Hypertensive Nephropathy. Medicina.

[51] Gerardo Yanowsky-Escatell F., Andrade-Sierra J., Pazarín-Villaseñor L., Santana-Arciniega C., De Jesús Torres-Vázquez E., Samuel Chávez-Iñiguez J., Ángel Zambrano-Velarde M., Martín Preciado-Figueroa F. (2020). The Role of Dietary Antioxidants on Oxidative Stress in Diabetic Nephropathy. Iran J. Kidney Dis..

[52] Radcliffe N.J., Seah J.M., Clarke M., MacIsaac R.J., Jerums G., Ekinci E.I. (2017). Clinical predictive factors in diabetic kidney disease progression. J. Diabetes Investig..

[53] Santoro D., Torreggiani M., Pellicanò V., Cernaro V., Messina R.M., Longhitano E., Siligato R., Gembillo G., Esposito C., Piccoli G.B. (2021). Kidney Biopsy in Type 2 Diabetic Patients: Critical Reflections on Present Indications and Diagnostic Alternatives. Int. J. Mol. Sci..

[54] Amatruda M., Gembillo G., Giuffrida A.E., Santoro D., Conti G. (2021). The Aggressive Diabetic Kidney Disease in Youth-Onset Type 2 Diabetes: Pathogenetic Mechanisms and Potential Therapies. Medicina.

[55] Giandalia A., Giuffrida A.E., Gembillo G., Cucinotta D., Squadrito G., Santoro D., Russo G.T. (2021). Gender Differences in Diabetic Kidney Disease: Focus on Hormonal, Genetic and Clinical Factors. Int. J. Mol. Sci..

[56] Bilous R.W., Gonzalez-Campoy J.M., Fradkin J.E., Mauer M., Molitch M.E., Narva A.S., Nelson R.G., Sharma K., Tuttle K.R., Rocco M.V. (2012). KDOQI Clinical Practice Guideline for Diabetes CKD: 2012 Update. Am. J. Kidney Dis..

[57] Gembillo G., Cernaro V., Salvo A., Siligato R., Laudani A., Buemi M., Santoro D. (2019). Role of Vitamin D Status in Diabetic Patients with Renal Disease. Medicina.

[58] Cernaro V., Loddo S., Macaione V., Ferlazzo V.T., Cigala R.M., Crea F., De Stefano C., Genovese A.R.R., Gembillo G., Bolignano D. (2020). RAS inhibition modulates kynurenine levels in a CKD population with and without type 2 diabetes mellitus. Int. Urol. Nephrol..

[59] Zelniker T.A., Wiviott S.D., Raz I., Im K., Goodrich E.L., Bonaca M.P., Mosenzon O., Kato E.T., Cahn A., Furtado R.H.M. (2019). SGLT2 inhibitors for primary and secondary prevention of cardiovascular and renal outcomes in type 2 diabetes: A systematic review and meta-analysis of cardiovascular outcome trials. Lancet.

[60] Bolignano D., Cernaro V., Gembillo G., Baggetta R., Buemi M., D’Arrigo G. (2017). Antioxidant agents for delaying diabetic kidney disease progression: A systematic review and meta-analysis. PLoS ONE.

[61] Wijesekara N., Chimienti F., Wheeler M.B. (2009). Zinc, a regulator of islet function and glucose homeostasis. Diabetes Obes. Metab..

[62] Prasad A.S., Bao B., Beck F.W., Kucuk O., Sarkar F.H. (2004). Antioxidant effect of zinc in humans. Free Radic. Biol. Med..

[63] Pompano L.M., Boy E. (2021). Effects of Dose and Duration of Zinc Interventions on Risk Factors for Type 2 Diabetes and Cardiovascular Disease: A Systematic Review and Meta-Analysis. Adv. Nutr..

[64] Xu J., Zhou Q., Liu G., Tan Y., Cai L. (2013). Analysis of serum and urinal copper and zinc in Chinese northeast population with the prediabetes or diabetes with and without complications. Oxidative Med. Cell. Longev..

[65] Al-Timimi D.J., Sulieman D.M., Hussen K.R. (2014). Zinc status in type 2 diabetic patients: Relation to the progression of diabetic nephropathy. J. Clin. Diagn. Res..

[66] Lin C.C., Shih C.T., Lee C.H., Huang Y.L. (2018). Changes in Trace Elements During Early Stages of Chronic Kidney Disease in Type 2 Diabetic Patients. Biol. Trace Elem. Res..

[67] Takao T., Yanagisawa H., Suka M., Yoshida Y., Onishi Y., Tahara T., Kikuchi T., Kushiyama A., Anai M., Takahashi K. (2022). Synergistic association of the copper/zinc ratio under inflammatory conditions with diabetic kidney disease in patients with type 2 diabetes: The Asahi Diabetes Complications Study. J. Diabetes Investig..

[68] Farvid M.S., Jalali M., Siassi F., Hosseini M. (2005). Comparison of the effects of vitamins and/or mineral supplementation on glomerular and tubular dysfunction in type 2 diabetes. Diabetes Care.

[69] Kadhim H.M., Ismail S.H., Hussein K.I., Bakir I.H., Sahib A.S., Khalaf B.H., Hussain S.A. (2006). Effects of melatonin and zinc on lipid profile and renal function in type 2 diabetic patients poorly controlled with metformin. J. Pineal Res..

[70] Parham M., Amini M., Aminorroaya A., Heidarian E. (2008). Effect of zinc supplementation on microalbuminuria in patients with type 2 diabetes: A double blind, randomized, placebo-controlled, cross-over trial. Rev. Diabet. Stud..

[71] El-Ashmony S.M.A., Morsi H.K., Abdelhafez A.M. (2012). Effect of zinc supplementation on glycemic control, lipid profile, and renal functions in patients with type II diabetes: A single blinded, randomized, placebo-controlled, trial. J Biol Agric Health.

[72] Narváez-Caicedo C., Moreano G., Sandoval B.A., Jara-Palacios M.Á. (2018). Zinc Deficiency among Lactating Mothers from a Peri-Urban Community of the Ecuadorian Andean Region: An Initial Approach to the Need of Zinc Supplementation. Nutrients.

[73] Fischer Walker C., Ezzati M., Black R. (2009). Global and regional child mortality and burden of disease attributable to zinc deficiency. Eur. J. Clin. Nutr..

[74] King J.C., Brown K.H., Gibson R.S., Krebs N.F., Lowe N.M., Siekmann J.H., Raiten D.J. (2015). Biomarkers of Nutrition for Development (BOND)-Zinc Review. J. Nutr..

[75] Brown K.H., Rivera J.A., Bhutta Z., Gibson R.S., King J.C., Lönnerdal B., Ruel M.T., Sandtröm B., Wasantwisut E., Hotz C. (2004). Assessment of the risk of zinc deficiency in populations and options for its control. Food Nutr. Bull..

[76] Mayo Clinic Laboratories Website. https://www.mayocliniclabs.com/test-catalog/overview/8620#Clinical-and-Interpretive.

[77] Wessells K.R., King J.C., Brown K.H. (2014). Development of a Plasma Zinc Concentration Cutoff to Identify Individuals with Severe Zinc Deficiency Based on Results from Adults Undergoing Experimental Severe Dietary Zinc Restriction and Individuals with Acrodermatitis Enteropathica. J. Nutr..

[78] Jameson S. (2000). Coeliac disease, insulin-like growth factor, bone mineral density, and zinc. Scand. J. Gastroenterol..

[79] Ojuawo A., Keith L. (2002). The serum concentrations of zinc, copper and selenium in children with inflammatory bowel disease. Cent. Afr. J. Med..

[80] Maxfield L., Shukla S., Crane J.S. Zinc Deficiency. https://www.ncbi.nlm.nih.gov/books/NBK493231/.

[81] Trumbo P., Yates A.A., Schlicker S., Poos M. (2001). Dietary reference intakes: Vitamin A, vitamin K, arsenic, boron, chromium, copper, iodine, iron, manganese, molybdenum, nickel, silicon, vanadium, and zinc. J. Am. Diet. Assoc..

[82] Matthew A.W., Kevin P.K., Wanda M.H., Colin G.R., Matthew A. (2013). Chapter 36—Nutritional Toxicologic Pathology. Wallig, Haschek and Rousseaux’s Handbook of Toxicologic Pathology.

[83] Al-Maroof R.A., Al-Sharbatti S.S. (2006). Serum zinc levels in diabetic patients and effect of zinc supplementation on glycemic control of type 2 diabetics. Saudi Med. J..

[84] Gunasekara P., Hettiarachchi M., Liyanage C., Lekamwasam S. (2011). Effects of zinc and multimineral vitamin supplementation on glycemic and lipid control in adult diabetes. Diabetes, Metabolic Syndrome and Obesity: Targets Ther..

[85] Fernández-Cao J.C., Warthon-Medina M., HMoran V., Arija V., Doepking C., Serra-Majem L., Lowe N.M. (2019). Zinc Intake and Status and Risk of Type 2 Diabetes Mellitus: A Systematic Review and Meta-Analysis. Nutrients.

[86] Luo Y.Y., Zhao J., Han X.Y., Zhou X.H., Wu J., Ji L.N. (2015). Relationship between serum zinc level and microvascular complications in patients with type 2 diabetes. Chin. Med. J..

[87] Jayawardena R., Ranasinghe P., Galappatthy P., Malkanthi R., Constantine G., Katulanda P. (2012). Effects of zinc supplementation on diabetes mellitus: A systematic review and meta-analysis. Diabetol. Metab. Syndr..

[88] Fischer P.W., Giroux A., L’Abbé M.R. (1984). Effect of zinc supplementation on copper status in adult man. Am. J. Clin. Nutr..

[89] Koi S., Arai K., Nogami A., Toma H., Yamamoto M., Miura O., Nagao T. (2020). Impaired hematopoiesis due to copper deficiency in a hemodialysis patient supplemented with zinc. Rinsho Ketsueki..

[90] Duncan A., Yacoubian C., Watson N., Morrison I. (2015). The risk of copper deficiency in patients prescribed zinc supplements. J. Clin. Pathol..

[91] Laouali N., MacDonald C.J., Shah S., El Fatouhi D., Mancini F.R., Fagherazzi G., Boutron-Ruault M.C. (2021). Dietary Copper/Zinc Ratio and Type 2 Diabetes Risk in Women: The E3N Cohort Study. Nutrients.

